# CCND1 as a Prognostic and Diagnostic Biomarker and the Impact of Its Epigenetic Alterations on Cancer Survival

**DOI:** 10.7759/cureus.65504

**Published:** 2024-07-27

**Authors:** Muhammed Y Taha, Noha O Mohamed, Lina G Alhaj, Issra Altayeb, Abeer Basheer, Shaymaa Idrees, Abdirahman M Said, Mohamed Alfaki

**Affiliations:** 1 Pharmaceutical Services, Almoosa Rehabilitation Hospital, Al Ahsa, SAU; 2 Medical Laboratory Sciences, A'Sharqiyah University, Ibra, OMN; 3 Pharmaceutical Services, Khartoum Oncology Hospital, Khartoum, SDN; 4 Faculty of Pharmacy, Al-Neelain University, Khartoum, SDN; 5 Pharmaceutical Services, Alzafer Hospital, Najran, SAU; 6 Faculty of Pharmacy, University of Khartoum, Khartoum, SDN; 7 Microbiology, University of Bosaso, Bosaso, SOM; 8 Research, Sidra Medicine, Doha, QAT

**Keywords:** genetic alterations, prognostic biomarker, immune infiltration, dna methylation, pan-cancer analysis, ccnd1

## Abstract

Background: Cyclin D1 (CCND1) plays a crucial role in cell cycle regulation and has been implicated in various cancers. As is well known, cancer is caused by the accumulation of detrimental variations in the genome. In this study, we shed light on the role of CCND1 in the diagnosis and progression of cancer and aimed to provide a comprehensive analysis of CCND1 across multiple cancer types, focusing on its expression, clinical correlations, DNA methylation status, prognostic implications, genetic alterations, and immune infiltration.

Methods: Gene expression analysis of CCND1 was conducted across 33 cancer types using the TIMER, GEPIA, and UALCAN databases. Clinical parameters were investigated to assess their correlations with CCND1 expression. Methylation analysis was performed using the UALCAN and GSCA databases to investigate the relationship between CCND1 promoter methylation and gene expression and their association with survival. Immune infiltration and survival analyses were performed to explore the prognostic implications of CCND1 expression in various cancers. Statistical tests, such as the Cox proportional hazards model and the Kaplan-Meier analysis, were used to assess survival outcomes. Additionally, genetic alteration analysis was performed using the cBioPortal database to examine the prevalence and types of CCND1 alterations across different cancer types.

Results: CCND1 expression was significantly elevated in 13 cancers compared to normal tissues, with distinct patterns observed across different cancer types. It is highly expressed in BLCA, CHOL, COAD, ESCA, GBM, HNSC, KIRC, PAAD, RRAD, READ, STAD, THCA, and UCEC. The investigation of clinical parameters revealed associations between CCND1 expression and factors such as age, gender, race, and cancer stage. The methylation analysis highlighted hypomethylation of CCND1 across the 13 selected cancer types. The survival analysis identified both favorable and unfavorable prognostic implications of CCND1 expression in different cancers and revealed that a high expression of CCND1 was associated with a poor prognosis in HNSC and PAAD, while a high expression of CCND1 was associated with a good prognosis in KIRC, STAD, THCA, and UCEC. In the immune infiltration analysis of various cancers, many statistically significant correlations were observed between the immune cell types and tumor purity. For example, in BLCA, neutrophils and dendritic cells showed statistically significant positive correlations and a negative correlation with macrophages. While in CHOL patients, none of the immune cell types showed a significant correlation. Similar statistical significance was observed in other cancer types, such as COAD, HNSC, GBM, KIRC, PAAD, PRAD, READ, and STAD, with different immune cell types. The genetic alteration analysis revealed that amplification was the predominant genetic alteration type in CCND1, with specific patterns observed in different cancer types.

Conclusion: The findings of this study provide valuable insights into the role of CCND1 in cancer diagnosis and progression, and its potential for targeted therapies. CCND1 could be used as a potential diagnostic biomarker for the COAD, ESCA, KIRC, READ, STAD, and THCA stages. Furthermore, CCND1 could be used as a potential prognostic biomarker for HNSC, KIRC, and PAAD. Also, the correlation between CCND1 methylation and expression could be used as a potential diagnostic and prognostic biomarker for ESCA, HNSC, and STAD.

## Introduction

Cancer is a leading cause of death worldwide [1]. It is an encompassing term for a diverse range of diseases affecting any bodily region, characterized by the rapid growth of abnormal cells exceeding normal boundaries. This leads to invasion of neighboring tissues and metastasis, the primary contributor to cancer-related mortality [2]. Cancer develops from genetic mutations within cells, leading to disruptions in their behavior such as increased division, impaired growth regulation, or faulty DNA repair. The effects depend on the type of gene mutation affecting proto-oncogenes, tumor suppressor genes, or DNA repair genes [3].

CCND1 (cyclin D1) is a protein-coding gene that belongs to a family called cyclins, which are a type of cell cycle regulators. Transitions through the cell cycle are driven by cyclins and cyclin-dependent kinases (CDKs) [4]. D cyclins, including cyclins D1, D2, and D3, form active complexes with either CDK4 or CDK6, which in turn phosphorylate the retinoblastoma protein (Rb) and drive G1 to S phase progression [5]. Cyclin D1, with its partner CDKs, regulates G1/S transition through Rb phosphorylation [6]. Small polypeptide inhibitors of CDK4/CDK6 efficiently block Rb phosphorylation in vivo. Moreover, Rb is also phosphorylated by cyclin E-CDK2 in the late G1 phase. The hyperphosphorylation of Rb triggers reduced affinity for E2F (transcription factors), thereby permitting E2F activation and transcription of client genes required for cell division [7].

In human tumors, the cyclin D1-CDK4 axis shows a high frequency of alterations, highlighting the importance of this pathway for tumor progression. With the recent advent of small molecule inhibitors of CDK4/CDK6, it is critical to discern key contributions of cyclin D1 with CDK-dependent and -independent effects in order to develop rational and successful therapeutic regimes [8]. The CCND1 gene is pivotal in controlling the advancement of the cell cycle and its proliferation. Irregularities in this gene have been connected to the emergence and advancement of multiple cancer forms, highlighting its significance as a focus for therapeutic strategies and ongoing investigations [9]. These studies indicate that altered CCND1 expression could promote carcinogenesis, and CCND1 plays a significant role in controlling the progression of the cell cycle and its proliferation. Despite this, no study to date has investigated the role of CCND1 as a diagnostic or prognostic biomarker in cancer. In addition, its potential for targeted therapies remains unclear.

In this study, we conducted a comprehensive pan-cancer analysis to determine CCND1's diagnostic function and prognostic significance in a number of cancer types. We investigated CCND1 expression, clinical correlations, DNA methylation status, prognostic implications, genetic alterations, and immune infiltration using patient data from different databases like TIMER, GEPIA, and UALCAN.

## Materials and methods

Gene expression analysis

We started our gene expression analysis using the TIMER database (https://cistrome.shinyapps.io/timer/, accessed on November 22, 2023), which is a comprehensive analytic web tool for cancer researchers to conveniently access the immunologic, clinical, and genomic features of tumors [10]. We used the module “DiffExp” to explore CCND1 expression across 33 distinct types of cancer. Our primary objective was to identify types demonstrating statistically significant differences in CCND1 expression, with strict criteria of a log fold change (logFC) of |1.5| and a p-value less than 0.05. Subsequently, we searched deeper into the significant types using both the GEPIA (http://gepia.cancer-pku.cn/, accessed on November 17, 2023) [11] and UALCAN (https://ualcan.path.uab.edu/, accessed on November 17, 2023) [12] databases, aiming to confirm and strengthen our findings. Furthermore, to enhance the reliability of our study, we specifically selected types of cancer that exhibited statistically significant modulation in CCND1 gene expression across at least two distinct databases. This step ensured a thorough and cross-validated exploration of CCND1 expression, thus enhancing the validity and relevance of our findings. 

Clinical parameter analysis in UALCAN

With a particular focus on examining the expression patterns of CCND1, our research utilized UALCAN to conduct a thorough gene expression analysis within the selected cancer types. This comprehensive investigation included important clinical parameters such as age, gender, weight, race, and cancer stage in addition to gene expression profiling. Our goal was to clarify any possible relationships between these clinicopathological parameters and the CCND1 expression profile by systematically including these clinical variables in our analytical methodology.

Methylation analysis

To understand the relationship between CCND1 gene upregulation and DNA methylation, we investigated the UALCAN database’s "TCGA module" (accessed on January 16, 2024) [12] to explore CCND1 promoter DNA methylation levels in the selected cancers to compare between tumors and normal tissues, as well as between normal and all cancer stages.

The CCND1 methylation profile across the selected cancer was further investigated using the GSCA database (https://guolab.wchscu.cn/GSCA/#/, accessed on March 18, 2024] [13]. Three modules were used to assess the methylation status. The differential methylation module was used to conduct methylation analysis for the tumor and normal samples.

The methylation and survival module established survival analysis for both high- and low-methylated groups. Clinical data from tumor samples were retrieved from the TCGA database, and the Cox proportional-hazards model was used to calculate the risk ratio (hazard ratio) of the high-methylated group in comparison to the low-methylated group. The log-rank test was used to determine whether the survival difference between the groups was statistically significant.

The methylation and expression module was applied to provide information about the correlation between methylation and mRNA expression of CCND1 in the selected cancers. The mRNA expression and methylation data were combined using TCGA barcodes. Spearman correlation analysis was used to determine the correlation between CCND1 m-RNA expression and methylation levels, and the p-value was adjusted using the false discovery rate (FDR).

Immune cell infiltration

We investigated the association between CCND1 expression and immune cell infiltration across the chosen 13 cancer types, utilizing data from the TIMER database's "gene module." Correlation coefficients and corresponding p-values were used to examine the correlation between CCND1 expression and infiltrating immune cells, including B cells, CD8+, CD4+, T cells, neutrophils, macrophages, and dendritic cells. Statistical significance was determined by p-values (≤ 0.05).

In the context of CCND1 as a potential prognostic biomarker, a positive and significant correlation (p-value < 0.05) indicates that higher CCND1 levels are associated with increased immune cell infiltration, suggesting a favorable prognostic biomarker. Conversely, a negative and significant correlation (p-value < 0.05) suggests that higher CCND1 levels are linked to decreased immune cell infiltration, indicating a potentially unfavorable prognostic biomarker. These findings were integrated with CCND1 expression data across individual cancer stages and survival analyses to comprehensively understand CCND1's prognostic potential in the selected cancer types.

Survival analysis

Survival Analysis Using GEPIA

In the context of the selected cancer types and focusing specifically on CCND1, survival analysis was performed using the GEPIA database. This study aimed to investigate the potential prognostic implications of CCND1 expression. Survival data, including hazard ratios and corresponding p-values, were extracted and analyzed to understand the correlation between CCND1 expression levels and patient survival outcomes. This analysis provided valuable insights into our broader investigation.

Cox Proportional Hazards Model Analysis

A key component of our survival analysis was the Cox proportional hazards model. We extracted survival, immune infiltrates in association with CCND1 expression, and clinical variables (age, gender, ethnicity, and cancer stages) using the TIMER dataset. The immune cell infiltrates, CCND1, and the selected significant tumors were applied to the "survival module" in TIMER. Considering 95% confidence intervals, the model evaluated hazard ratios, coefficients, and p-values to estimate the risk of mortality. The findings were smoothly linked with past correlation data, providing a complete picture of CCND1's prospective potential in the cancer types studied.

Kaplan-Meier Plot Analysis

Additionally, we examined the impact of CCND1 on patient survival in significant cancer types using the Kaplan-Meier plotter (https://kmplot.com/analysis/, checked on December 3, 2023) [14]. By gathering survival data from GEPIA, UALCAN, and TIMER, we created survival curves to visualize how CCND1 expression relates to patient outcomes over time. By comparing these curves and using statistical tests, we explored whether CCND1 had a significant effect on survival. This analysis offered a clearer picture of CCND1's potential as a prognostic biomarker in the studied cancers.

Genetic alteration analysis

In this study, genomic data for the CCND1 gene was obtained from publicly available datasets using cBioPortal (https://www.cbioportal.org/) [15]. The “oncoprint module” was employed to visualize and interpret the genetic landscape of CCND1, showcasing patterns of missense mutations, amplifications, and deletions across diverse cancer samples. The cancer types summary module was utilized to categorize and summarise CCND1 alterations based on specific cancer types, providing insights into the distribution of mutations across different malignancies.

mRNA expression levels of CCND1 were analyzed across the dataset to understand how genetic alterations affect transcription. A comparison of expression with specific mutations aimed to discern the influence of genetic changes on CCND1. Mutation frequency plots were generated to illustrate the occurrence of driver mutations and variants of uncertain significance (VUS) in CCND1, offering a detailed examination of the mutation patterns. Additionally, the comparison/survival module in cBioPortal was used to assess the clinical relevance of CCND1 alterations, generating Kaplan-Meier survival curves to compare survival rates between groups with and without CCND1 mutations. Statistical analyses were performed to quantify and analyze observed genetic alterations and survival outcomes.

Validation

We used the GEO database (https://www.ncbi.nlm.nih.gov/geo/) [16] to confirm CCND1 expression in two carcinomas (HNSC and PAAD). We searched for HNSC and PAAD studies, applied the Homo sapiens filter, and selected the study type: expression profiling by array. Next, we searched for a dataset that included gene expression (mRNA expression) in tumor and normal tissues. As a result, we divided the samples into two categories: normal and tumor samples. The study was then examined using GEO2R, an analysis tool that compares two or more groups of GEO samples to determine their substantial differentiation across experimental circumstances. Furthermore, the data from the GEO database was copied to the website http://bioinformatics.com.cn [17] to visualize, specifically CCND1 gene expression, using the volcano plot. 

Additionally, CCND1 expression in HNSC, KIRC, PAAD, STAD, THCA, and UCEC was validated using UCSC Xena (https://xenabrowser.net/) [18]. GTEX represents normal tissue samples, whereas TCGA represents cancer tissues. The data were plotted as volcano plots using Anaconda Navigator (Anaconda, Inc., Austin, Texas, United States), Jupyter 6.5.4 (Project Jupyter, New York City, New York, United States), and Python3 (Python Software Foundation, Wilmington, Delaware, United States), and logFC and adjusted p-values were used to plot. 

Statistical analysis

The primary statistical test in our investigation was the adjusted p-value. The Wilcoxon test was used to calculate the statistical significance of the TIMER bioinformatics data, which is shown by the number of stars (*: p-value < 0.05; **: p-value <0.01; ***: p-value < 0.001). For the GEPIA database boxplots of expression and stage plots, the cutoff was set at logFC = 1.5 and q-value = 0.05, while a 95% confidence interval was used for the survival plot. Regarding survival analysis, Kaplan-Meier analysis, the log-rank test, and the Cox regression test were used, and results with a 95% confidence interval and a p-value of less than 0.05 were considered statistically significant. 

## Results

CCND1 expression pan-cancer analysis

**Figure 1:**
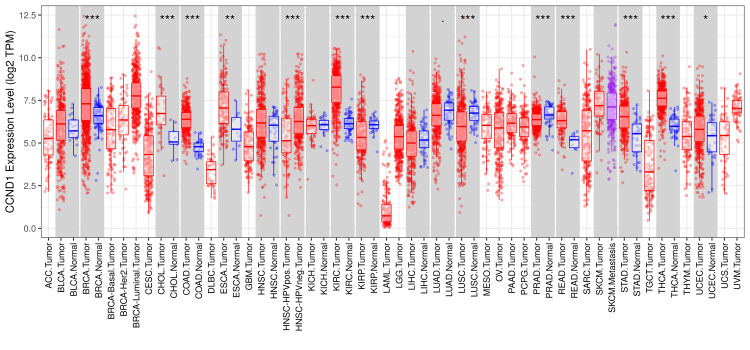
CCND1 expression profile across different cancer types using the TIMER database. Distributions of CCND1 expression levels are displayed using box plots; red box plots mean that CCND1 is up-regulated, and blue box plots mean it is down-regulated (*: p-value < 0.05; **: p-value <0.01; ***: p-value <0.001).

According to the results from the TIMER database, the CCND1 level was significantly higher in most tumors, such as BLCA, CHOL, COAD, ESCA, HNSC, KIRC, KIRP, LUSC, PAAD, PRAD, READ, STAD, THCA, and UCEC, versus adjacent normal tissues (Figure 1).

**Figure 2:**
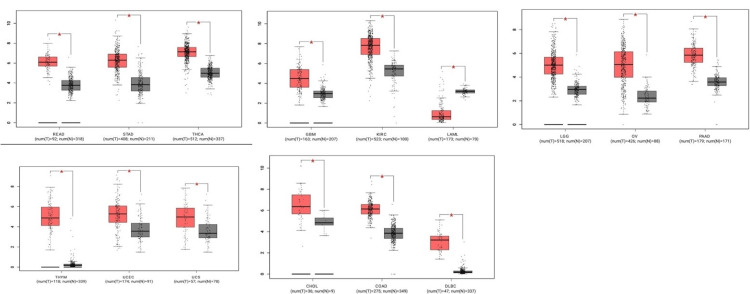
Gene expression among the significant cancer types using the GEPIA database. Each box plot represents the expression of CCND; red box plots refer to up-regulation, and grey box plots represent down-regulation.

**Figure 3:**
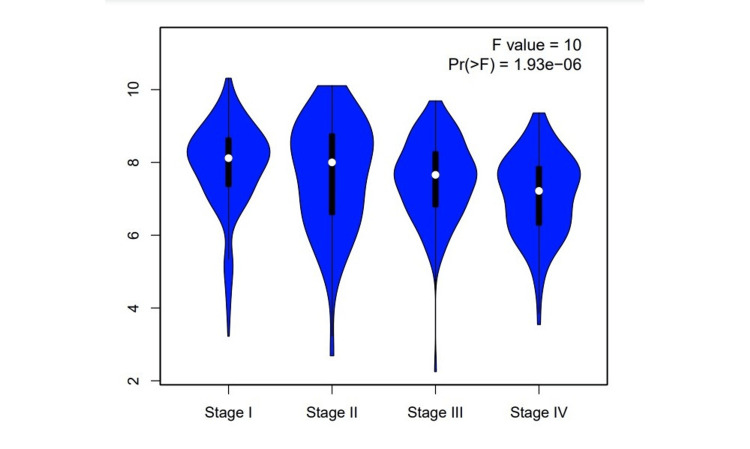
Stage analysis of CCND1 in KIRC from GEPIA

According to the GEPIA database, CCND1 expression level has been significantly higher in these types of cancer: CHOL, COAD, DLBC, GBM, KIRC, LAML, LGG, OV, PAAD, READ, STAD, THCA, THYM, UCEC, and UCS, as shown in Figure 2. The stage analysis showed that the accounted significant difference between normal and tumor samples has occurred in kidney renal clear cell carcinoma, with an F value of 10 demonstrating the ratio of the variance between groups to the variance within groups in the analysis (Figure 3). A higher F value suggests more significant differences between groups. The F-test in the analysis of KIRC showed a significant result (p-value 1.93e−06), indicating that there were statistically significant differences between the compared groups.

**Figure 4:**
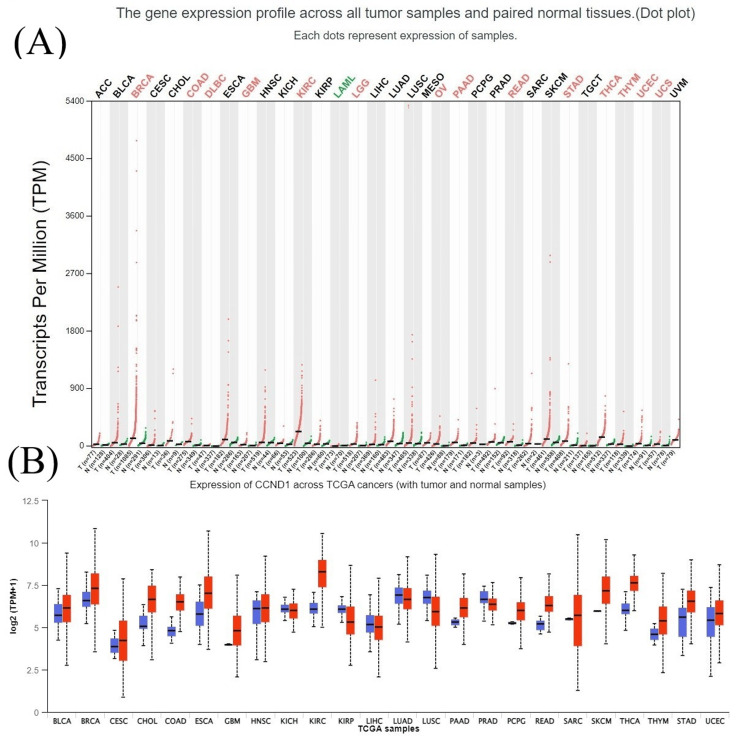
CCND1 expression profile across different human tumors and normal tissues using different databases. (A) CCND1 expression profile among different types of cancer using the GEPIA database. Each dot represents the expression of the samples; red dots mean high expression, while green dots mean low expression. (B) Expression profile of CCND1 gene across different cancer types extracted from the UALCAN database. Distributions of CCND1 expression levels displayed using box plots; red box plots mean that CCND1 is up-regulated, and blue box plots mean it is down-regulated.

The results from the UALCAN database also revealed that CCND1 expression was significantly higher in most cancer types (BLCA, BRCA, CHOL, COAD, ESCA, GBM, HNSC, KIRC, PAAD, READ, STAD, PCPG, THCA, THYM, UCEC, and UVM) which matched with TIMER and GEPIA database results (Figure 4).

Clinical parameter analysis in UALCAN

**Figure 5:**
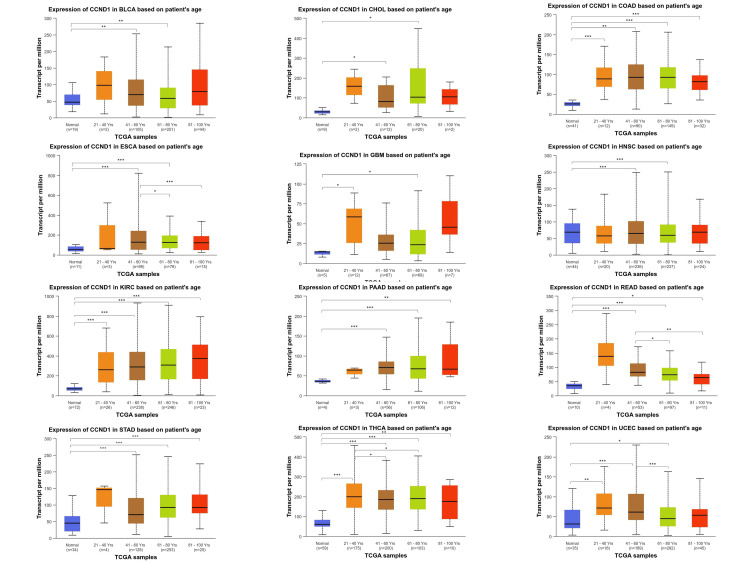
CCND1 expression across 12 cancer types (BLCA, CHOL, COAD, ESCA, GBM, HNSC, KIRC, PAAD, READ, STAD, THCA, and UCEC) based on age groups: 21-40 years, 41-60 years, 61-80 years, and 81-100 years using the UALCAN database. Statistical significance is annotated by the number of stars (*: p-value < 0.05; **: p-value <0.01; ***: p-value <0.001).

We conducted further analysis to evaluate the correlation between CCND1 expression and clinical and pathological characteristics in the UALCAN database, including age classification (21-40; 41-60; 61-80; and 81-100 years) we observed that CCND1 was over-expressed in 12 tumors, other than PRAD, compared to the expression in healthy tissues according to data presented in Figure 5 and Table 1. Interestingly, in both ESCA and READ, the CCND1 expression level was significantly higher in the age group 41-61 years as compared to the age groups 61-80 years and 81-100 years. The CCND1 gene expression in THCA was the highest in the age group 21-40 years compared to the other age groups.

**Table 1:** CCND1 expression among the significant cancers integrated with clinical parameters using the UALCAN database.

Types of cancer	Gene expression based on patients' gender	p-value	Gene expression based on patients' age	p-value	Gene expression based on patients' weight	p-value	Gene expression based on individual cancer stages	p-value	Gene expression based on patients' race	p-value
Bladder urothelial carcinoma	Normal-vs-Male	9.83E-04	Normal-vs-Age(41-60 years)	1.82E-03	Normal-vs-Normal Weight	7.82E-03	Normal-vs-Stage2	1.18E-03	Normal-vs-Caucasian	7.55E-04
	Normal-vs-Female	4.26E-03	Normal-vs-Age(61-80 years)	3.86E-03	Normal-vs-Extreme Weight	1.21E-02	Normal-vs-Stage3	2.81E-02	Normal-vs-Asian	1.35E-02
	-	-	-	-	Normal-vs-Obese	1.76E-02	Normal-vs-Stage4	9.18E-03	-	-
Cholangiocarcinoma	Normal-vs-Male	1.08E-03	Normal-vs-Age(41-60 years)	2.00E-02	Normal-vs-Obese	1.24E-02	Normal-vs-Stage1	1.92E-03	Normal-vs-Caucasian	3.40E-03
	Normal-vs-Female	3.42E-02	Normal-vs-Age(61-80 years)	1.62E-02	-	-	Normal-vs-Stage4	1.12E-02	Normal-vs-African American	2.20E-05
	-	-	-	-	-	-	Stage1-vs-Stage4	3.98E-02	-	-
Colon adenocarcinoma	Normal-vs-Male	1.62E-12	Normal-vs-Age(21-40 years)	1.01E-04	Normal-vs-Normal Weight	<1E-12	Normal-vs-Stage1	2.94E-10	Normal-vs-Caucasian	1.62E-12
	Normal-vs-Female	<1E-12	Normal-vs-Age(41-60 years)	<1E-12	Normal-vs-Extreme Weight	1.62E-12	Normal-vs-Stage2	1.62E-12	Normal-vs-African American	1.11E-15
	-	-	Normal-vs-Age(61-80 years)	1.62E-12	Normal-vs-Obese	2.22E-15	Normal-vs-Stage3	<1E-12	Normal-vs-Asian	5.44E-04
	-	-	Normal-vs-Age(81-100 years)	1.48E-08	Normal-vs-Extreme Obese	1.00E-03	Normal-vs-Stage4	4.19E-11	-	-
Esophageal carcinoma	Normal-vs-Male	5.03E-08	Normal-vs-Age(41-60 years)	2.11E-06	Normal-vs-Normal Weight	4.04E-07	Normal-vs-Stage1	4.95E-02	Normal-vs-Caucasian	2.42E-05
	-	-	Normal-vs-Age(61-80 years)	2.50E-05	Normal-vs-Extreme Weight	2.36E-03	Normal-vs-Stage2	4.32E-05	Normal-vs-Asian	1.01E-04
	-	-	Age(41-60 years)-vs-Age(61-80 years)	1.02E-02	Normal-vs-Obese	2.19E-02	Normal-vs-Stage3	4.15E-05	-	-
	-	-	Age(41-60 years)-vs-Age(81-100 years)	6.57E-04	Normal-vs-Extreme Obese	2.46E-02	Normal-vs-Stage4	2.02E-02	-	-
	-	-	-	-	Normal Weight-vs-Extreme Weight	5.10E-03	-	-	-	-
	-	-	-	-	Normal Weight-vs-Obese	1.58E-03	-	-	-	-
	-	-	-	-	Normal Weight-vs-Extreme Obese	9.00E-03	-	-	-	-
Head and neck squamous cell carcinoma	Normal-vs-Male	5.25E-09	Normal-vs-Age(41-60 years)	1.66E-05	-	-	Normal-vs-Stage4	1.24E-08	Normal-vs-Caucasian	7.51E-09
	Normal-vs-Female	5.82E-03	Normal-vs-Age(61-80 years)	1.31E-06	-	-	Stage2-vs-Stage4	7.04E-04	Normal-vs-African American	1.78E-02
	-	-	-	-	-	-	Stage3-vs-Stage4	1.13E-02	Caucasian-vs-Asian	1.20E-03
	-	-	-	-	-	-	-	-	African American-vs-Asian	3.31E-02
Glioblastoma multiforme	Normal-vs-Female	1.10E-02	Normal-vs-Age(21-40 years)	3.51E-02	-	-	-	-	-	-
	-	-	Normal-vs-Age(61-80 years)	1.06E-02	-	-	-	-	-	-
Kidney renal clear cell carcinoma	Normal-vs-Male	1.62E-12	Normal-vs-Age(21-40 years)	1.47E-06	-	-	Normal-vs-Stage1	1.62E-12	Normal-vs-Caucasian	1.62E-12
	Normal-vs-Female	1.62E-12	Normal-vs-Age(41-60 years)	<1E-12	-	-	Normal-vs-Stage2	7.99E-10	Normal-vs-African American	1.78E-11
	Male-vs-Female\	2.53E-06	Normal-vs-Age(61-80 years)	1.62E-12	-	-	Normal-vs-Stage3	<1E-12	Normal-vs-Asian	1.07E-02
	-	-	Normal-vs-Age(81-100 years)	6.10E-07	-	-	Normal-vs-Stage4	1.73E-14	-	-
	-	-	-	-	-	-	Stage1-vs-stage3	6.28E-05	-	-
	-	-	-	-	-	-	Stage1-vs-Stage4	3.44E-13	-	-
	-	-	-	-	-	-	Stage1-vs-Stage5	7.57E-04	-	-
	-	-	-	-	-	-	Stage1-vs-Stage6	2.19E-03	-	-
Pancreatic adenocarcinoma	Normal-vs-Male	1.62E-09	Normal-vs-Age(41-60 years)	1.11E-06	-	-	-	-	Normal-vs-Caucasian	6.19E-10
	Normal-vs-Female	6.80E-08	Normal-vs-Age(61-80 years)	3.33E-09	-	-	Normal-vs-Stage2	4.17E-10	Normal-vs-African American	4.46E-02
	-	-	Normal-vs-Age(81-100 years)	3.12E-03	-	-	-	-	Normal-vs-Asian	4.36E-03
Prostate adenocarcinoma	-	-	-	-	-	-	-	-	Caucasian-vs-African American	2.85E-02
	-	-	-	-	-	-	-	-	-	-
Rectum adenocarcinoma	Normal-vs-Male	3.26E-11	Normal-vs-Age(41-60 years)	1.41E-09	Normal-vs-Normal Weight	1.00E-04	Normal-vs-Stage1	3.13E-07	Normal-vs-Caucasian	5.57E-12
	Normal-vs-Female	1.26E-09	Normal-vs-Age(61-80 years)	2.47E-09	Normal-vs-Extreme Weight	4.28E-07	Normal-vs-Stage2	2.46E-08	Normal-vs-African American	1.66E-03
	-	-	Normal-vs-Age(81-100 years)	1.41E-02	Normal-vs-Obese	3.18E-02	Normal-vs-Stage3	2.88E-08	-	-
	-	-	Age(41-60 years)-vs-Age(61-80 years)	4.32E-02	-	-	Normal-vs-Stage4	1.23E-06	-	-
	-	-	Age(41-60 years)-vs-Age(81-100 years)	6.46E-03	-	-			-	-
Stomach adenocarcinoma	Normal-vs-Male	9.51E-13	Normal-vs-Age(41-60 years)	4.23E-05	-	-	Normal-vs-Stage1	7.24E-03	Normal-vs-Caucasian	5.67E-12
	Normal-vs-Female	1.30E-09	Normal-vs-Age(61-80 years)	9.78E-14	-	-	Normal-vs-Stage2	1.57E-07	Normal-vs-African American	2.50E-02
	Male-vs-Female	2.89E-02	Normal-vs-Age(81-100 years)	1.17E-04	-	-	Normal-vs-Stage3	8.59E-11	Normal-vs-Asian	1.21E-04
	-	-	-	-	-	-	Normal-vs-Stage4	3.23E-04	-	-
	-	-	-	-	-	-	-	-	-	-
Thyroid carcinoma	Normal-vs-Male	1.60E-12	Normal-vs-Age(21-40 years)	1.62E-12	-	-	Normal-vs-Stage1	1.62E-12	Normal-vs-Caucasian	<1E-12
	Normal-vs-Female	1.60E-12	Normal-vs-Age(41-60 years)	<1E-12	-	-	Normal-vs-Stage2	1.73E-10	Normal-vs-African American	9.54E-12
	-	-	Normal-vs-Age(61-80 years)	1.62E-12	-	-	Normal-vs-Stage3	<1E-12	Normal-vs-Asian	1.95E-12
	-	-	Normal-vs-Age(81-100 years)	6.90E-03	-	-	Normal-vs-Stage4	7.77E-16	-	-
	-	-	Age(21-40 years)-vs-Age(41-60 years)	2.30E-02	-	-	-	-	-	-
	-	-	Age(21-40 years)-vs-Age(61-80 years)	2.56E-02	-	-	-	-	-	-
Uterine corpus endometrial carcinoma	-	-	Normal-vs-Age(21-40 years)	5.54E-03	Normal-vs-Normal Weight	1.78E-03	Normal-vs-Stage1	1.54E-03	Normal-vs-Caucasian	2.60E-04
	-	-	Normal-vs-Age(41-60 years)	2.84E-06	Normal-vs-Extreme Weight	7.24E-03	Normal-vs-Stage2	2.69E-02	Normal-vs-African American	2.40E-02
	-	-	Normal-vs-Age(61-80 years)	2.64E-02	Normal-vs-Obese	2.86E-03	Normal-vs-Stage3	3.63E-04	Normal-vs-Asian	1.38E-02
	-	-	Age(41-60 years)-vs-Age(61-80 years)	3.23E-04	Normal-vs-Extreme Obese	3.68E-03	-	-	-	-

**Figure 6:**
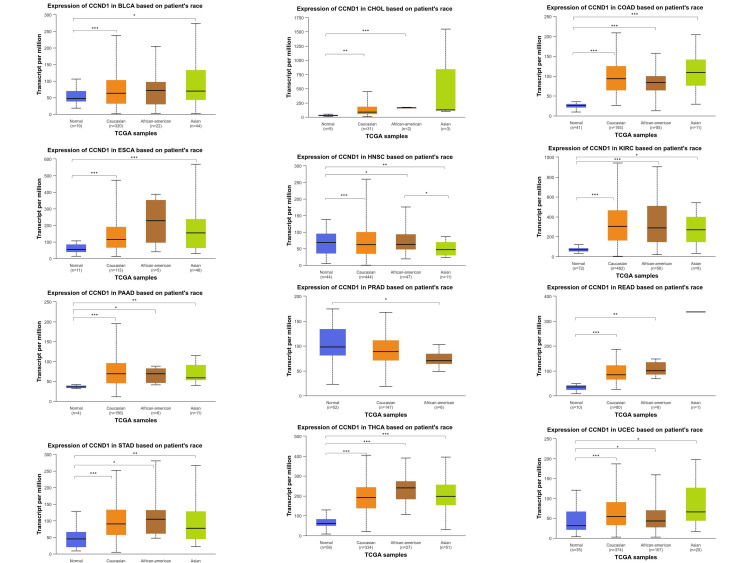
CCND1 expression in BLCA, CHOL, COAD, ESCA, HNSC, KIRC, PAAD, PRAD, READ, STAD, THCA, and UCEC patients according to patients' race (African American, Caucasian, and Asian) compared to normal sample using the UALCAN database. *p < 0.05, **p < 0.01, ***p < 0.001, and ****p<0.0001.

The CCND1 gene expression was highest in the Caucasian population and the lowest was observed in the Asian population. The gene expression was compared between normal sample tissues and three different racial categories: Caucasian, Asian, and African American. As seen in Figure 6, the variation in CCND1 expression across these cancers was clearly manifested.

**Figure 7:**
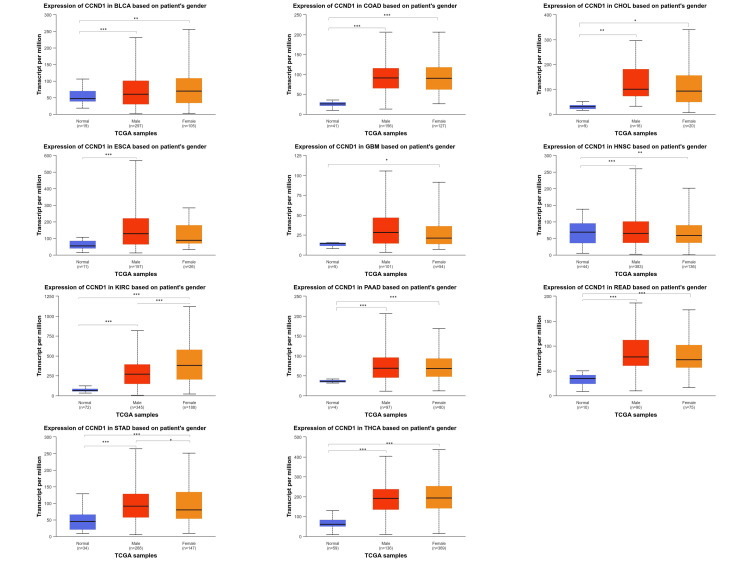
CCND1 expression across 11 cancer types (BLCA, CHOL, COAD, ESCA, GBM, HNSC, KIRC, PAAD, READ, STAD, and THCA) based on patients' gender (male and female) using the UALCAN database. *p < 0.05, **p < 0.01, ***p < 0.001, and ****p<0.0001.

For the gender parameters (male and female), we saw that CCND1 expression in BLCA, CHOL, COAD, HNSC, PAAD, READ, and THCA was upregulated in both male and female samples when compared to normal samples for each gene but the log fold change between male and female samples was not statistically significant. Nevertheless, the differentiation between female and male samples in relation to the manifestation of the disease demonstrated that female samples had significantly higher levels of CCND1 expression than males in KIRC and STAD. Whereas for CCND1, the relative gene expression in GBM (p = 1. 10E-02) was found to be enriched in females as compared to normal samples, for the ESCA (p = 5. 03E-08), relative gene expression was enriched for male samples as compared to normal samples (Figure 7 and Table 1 ). 

**Figure 8:**
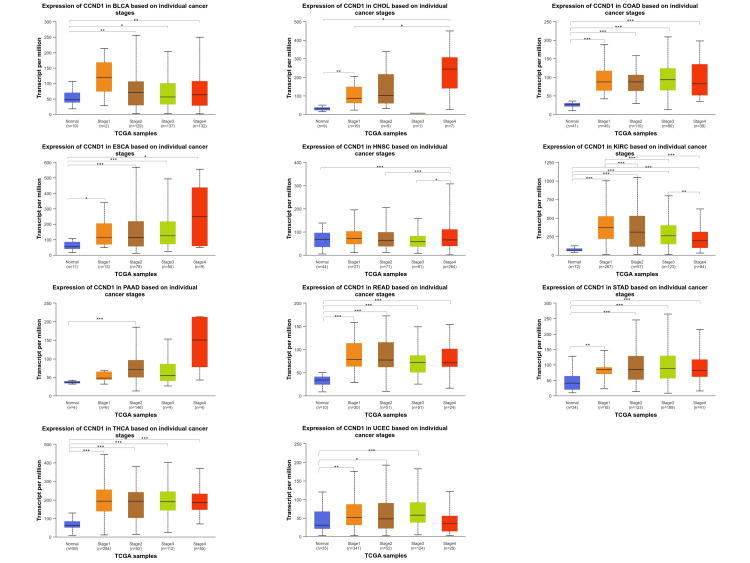
CCND1 expression according to the individual cancer stages (stages 1, 2, 3, and 4) across BLCA, CHOL, COAD, ESCA, HNSC, KIRC, PAAD, READ, STAD, THCA, and UCEC patients using the UALCAN database. Statistical significance is annotated by the number of stars (*: p-value < 0.05; **: p-value <0.01; ***: p-value <0.001).

Among various cancer types (BLCA, CHOL, COAD, ESCA, HNSC, KIRC, PAAD, READ, STAD, THCA, and UCEC), the CCND1 gene expression exhibited statistically significant differences when comparing normal tissue expression with stages 1 through 4 (Figure 8 and Table 1).

**Figure 9:**
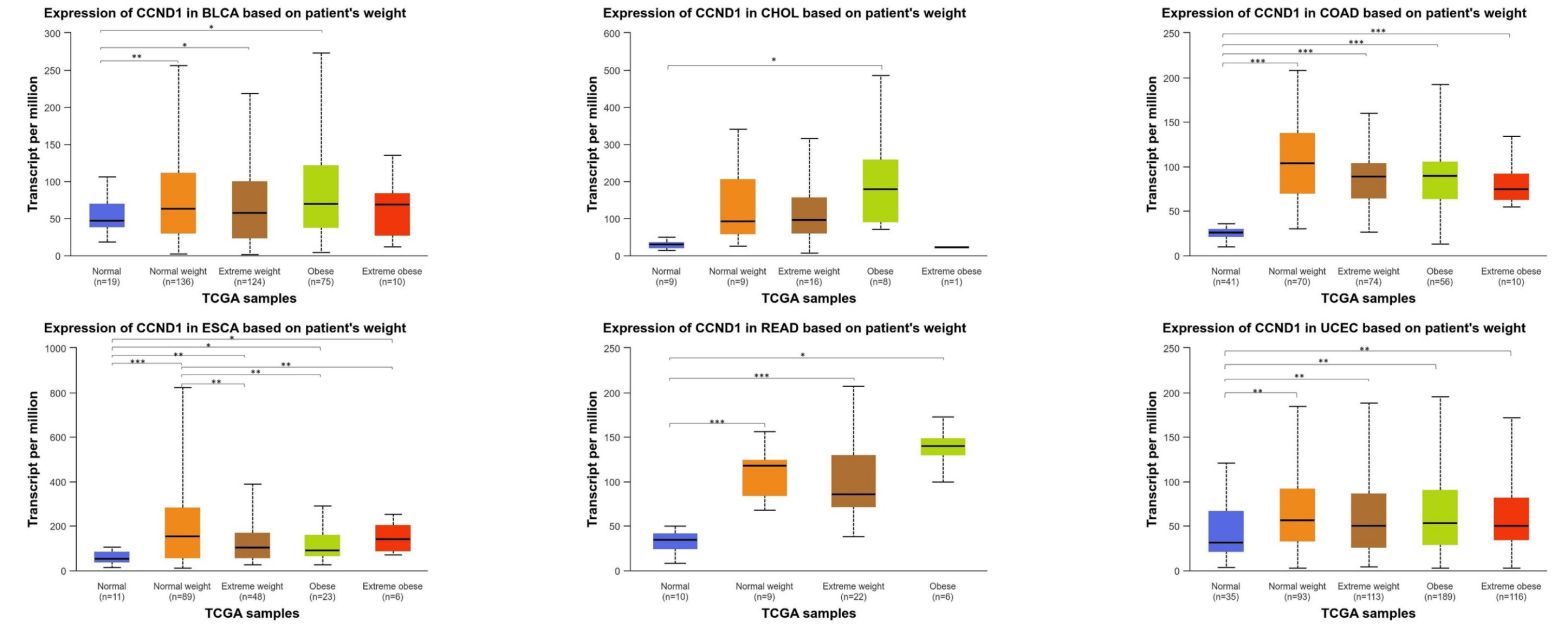
CCND1 expression in BLCA, CHOL, COAD, ESCA, READ, and UCEC patients according to patients' weight using the UALCAN database. Weight groups are grouped according to BMI into normal weight, extreme weight, obese, and extremely obese. *p < 0.05, **p < 0.01, ***p < 0.001, and ****p<0.0001.

Across various types of cancer (BLCA, CHOL, COAD, ESCA, READ, and UCEC), the CCND1 gene expression was statistically significant when comparing normal tissue samples with normal weight, extreme weight, obese, and extremely obese patients' samples with varying degrees of significance. These findings illustrated in Figure 9 and Table 1 revealed diverse significant correlations between genetic expression and clinical parameters in these types of cancers.

Methylation analysis

**Figure 10:**
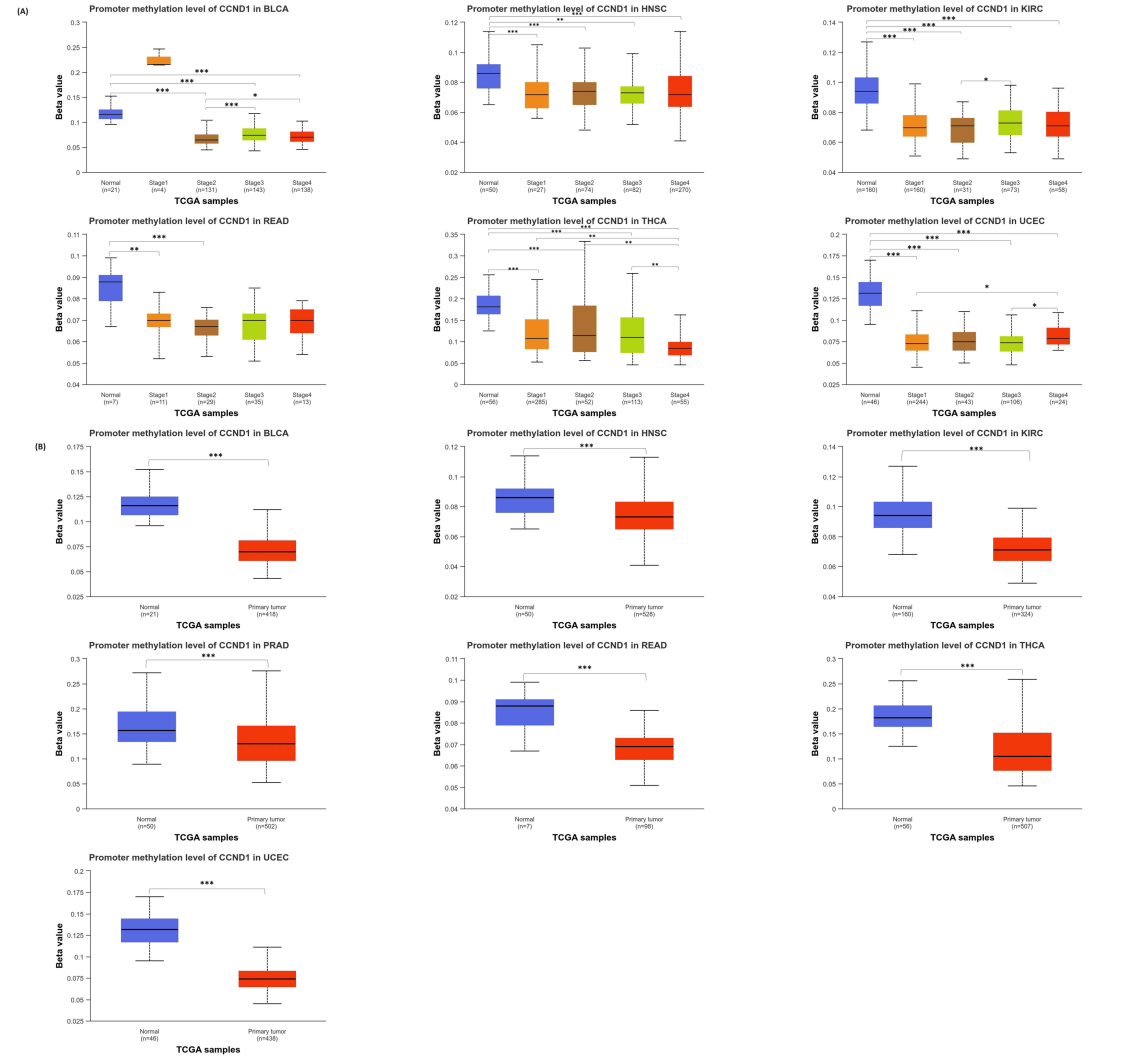
Using the UALCAN database, (A) promoter DNA methylation of CCND1 comparison between normal and tumor samples and (B) promoter DNA methylation of CCND1 in normal and tumor samples based on individual cancer stages. Statistical significance is annotated by the number of stars (*: p-value < 0.05; **: p-value <0.01; ***: p-value <0.001).

The results from the UALCAN database comparing cancer with normal tissues showed that the CCND1 promoter was hypomethylated in BLCA, HNSC, KIRC, READ, THCA, and UCEC (Figure 6). The p-value of promoter methylation of CCND1 in HNSC, KIRC, THCA, and UCEC was statistically significant between normal and all stages. In PAAD, the differences were statistically significant only between normal and stages 2 and 3. While in BLCA normal vs stages 2, 3, and 4 were statistically significant. Promoter methylation of CCND1 between BLCA and KIRC stages was statistically significant between stages 2 and 3. In addition, the BLCA stages 2 and 4 were statistically different. THCA and UCEC stages 1 and 3 were statistically significant. Moreover, THCA revealed differences between stages 2 and 4, as shown in Figure 10.

**Figure 11:**
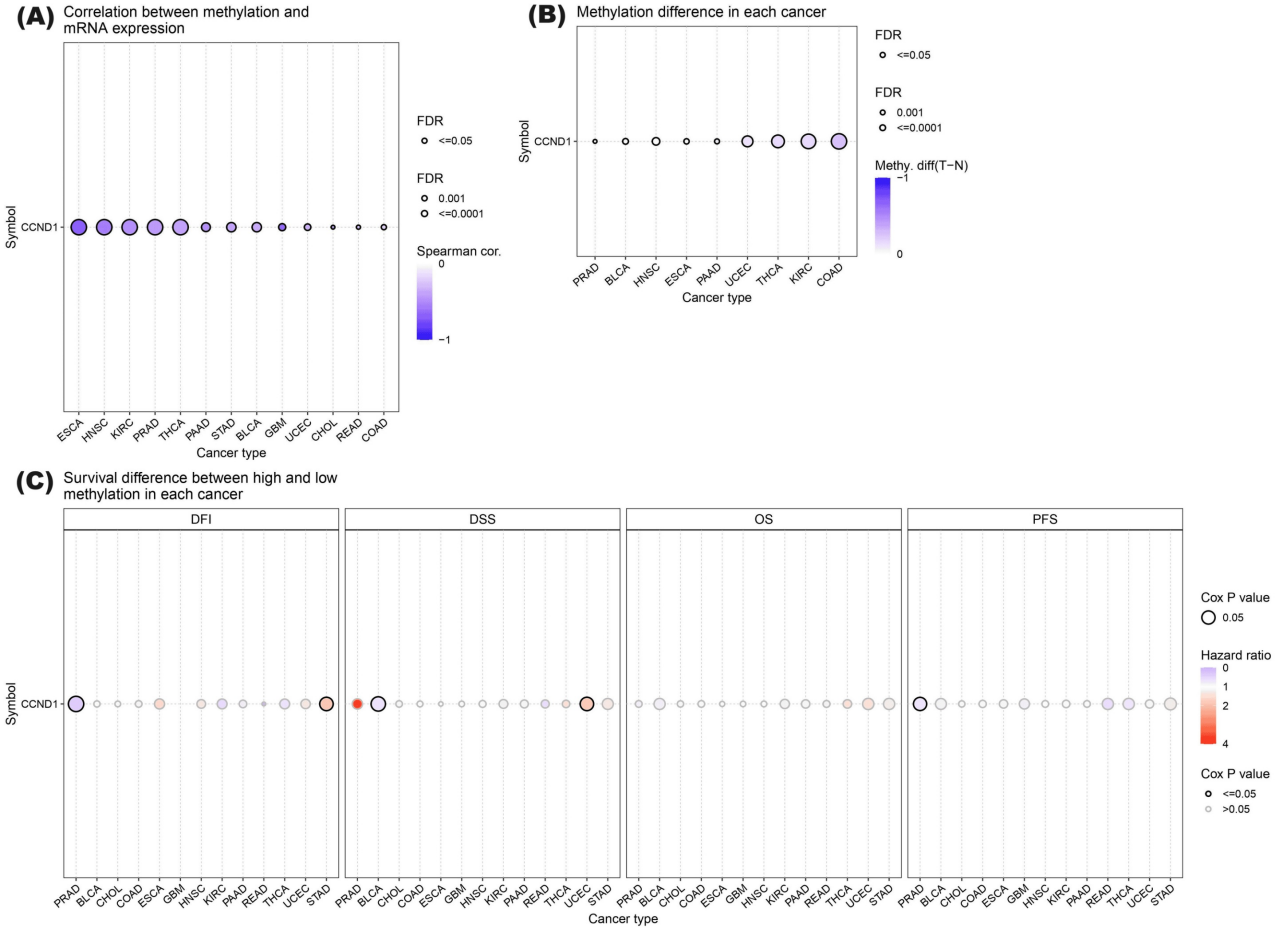
CCND1 methylation status across the significant selected cancers using the GSCA database. (A) Correlation between methylation levels at CpG sites and the expression of the CCND1 gene across various cancer types. (B) Differential methylation of the CCND1 gene in each cancer. (C) Survival difference (disease-free survival, disease-specific interval, overall survival, and progression-free survival) between higher and lower methylation groups in the specific cancers.

Furthermore, the methylation status of a single CpG island and the correlation between methylation status and survival probability in different human cancers were analyzed using the GSCA database (Figure 11 and Table 2). The results indicated a consistent negative correlation between methylation levels at these CpG sites: cg03040489, cg04717045, cg04717045, cg03040489, cg05164185, cg12266049, cg09637363, cg26399164, cg00953256, cg18773844 and cg26399164, and CCND1 expression across various cancer types. This suggests that hypermethylation of these sites may be associated with decreased expression of CCND1, which is a key regulator of cell cycle progression. Differential methylation of CCND1 has been observed in various cancers, with consistent downregulation across several types including BLCA, BRCA, COAD, ESCA, HNSC, KIRC, LUAD, PAAD, PRAD, THCA, and UCEC. The lower methylation of CCND1 is associated with poor prognosis in several cancers. In BLCA and PRAD, lower methylation levels are linked to a higher risk of death, progression, and disease recurrence. In contrast, in STAD and UCEC, higher methylation levels are linked to an increased risk of disease recurrence and death. These findings highlight the importance of CCND1 methylation status as a prognostic indicator in these cancers.

**Table 2:** Detailed information about the correlations between methylation and mRNA expression, the methylation difference between tumor and normal samples, and the overall survival difference between higher and lower methylation groups of CCND1 in the selected 13 cancers.

Cancer type	Differential methylation logFC	FDR	Survival type	Log-rank P	Hazard ratio	Correlation between CCND1 expression and methylation	FDR
BLCA	-0.02	9.09E-05	OS	0.16	0.81	-0.36	6.91E-14
			PFS	0.18	0.84		
			DSS	0.02	0.65		
			DFS	0.82	1.08		
CHOL	-0.26	1.77E-52	OS	0.81	0.89	-0.46	0.005
			PFS	0.81	0.90		
			DSS	0.78	0.86		
			DFS	0.89	1.11		
COAD			OS	0.76	0.93	-0.19	0.001
			PFS	0.67	0.92		
			DSS	0.87	0.94		
			DFS	0.76	1.17		
ESCA	-0.04	0.0002	OS	0.94	0.98	-0.69	0
			PFS	0.50	1.14		
			DSS	0.99	1.00		
			DFS	0.26	1.59		
GBM			OS	0.90	1.03	-0.64	7.55E-07
			PFS	0.26	0.81		
			DSS	0.89	1.03		
HNSC	-0.01	1.56E-08	OS	0.88	1.02	-0.55	0
			PFS	0.75	1.04		
			DSS	0.72	0.94		
			DFS	0.45	1.33		
KIRC	-0.16	1.91E-46	OS	0.37	1.19	-0.48	0
			PFS	0.64	1.08		
			DSS	0.43	1.21		
			DFS	0.29	0.55		
PAAD	-0.09	0.0007	OS	0.50	0.87	-0.48	5.13E-12
			PFS	0.77	0.95		
			DSS	0.56	0.88		
			DFS	0.62	0.81		
PRAD	-0.02	0.002	OS	0.75	0.81	-0.43	0
			PFS	0.05	0.67		
			DSS	0.22	3.64		
			DFS	0.01	0.37		
READ			OS	0.73	1.19	-0.29	0.005
			PFS	0.15	0.58		
			DSS	0.54	0.59		
			DFS	0.10	0.00		
STAD			OS	0.16	1.26	-0.38	5.64E-14
			PFS	0.11	1.26		
			DSS	0.17	1.32		
			DFS	0.04	1.87		
THCA	-0.16	2.03E-32	OS	0.47	1.43	-0.39	0
			PFS	0.15	0.69		
			DSS	0.63	1.44		
			DFS	0.29	0.66		
UCEC	-0.12	1.09E-21	OS	0.15	1.41	-0.33	1.08E-05
			PFS	0.51	1.13		
			DSS	0.04	1.81		
			DFS	0.35	1.33		

Immune infiltration analysis

**Figure 12:**
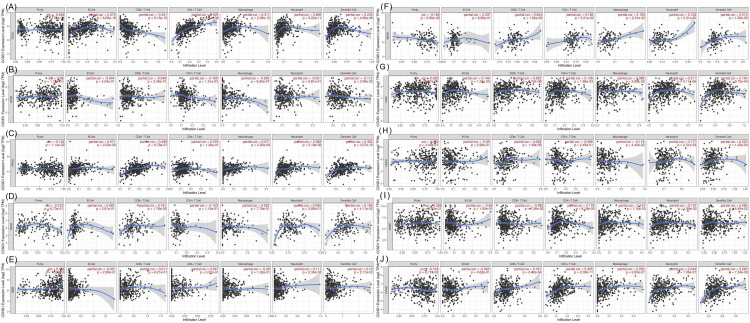
The association between CCND1 expression and immune infiltration levels using the TIMER database. (A-E) Correlation between CCND1 expression and the immune cells (B cells, CD8+, CD4+, T cells, neutrophils, macrophages, and dendritic cells) of THCA, STAD, PRAD, PAAD, and GBM. (F-J) Correlation between CCND1 expression and the immune cells (B cells, CD8+, CD4+, T cells, neutrophils, macrophages, and dendritic cells) of READ, KIRC, UCEC, HNSC, and COAD.

The immune cell infiltration analysis results from the TIMER database are shown in Figure 12. KIRC, PRAD, and THCA had a positive correlation with B cell, the correlation with KIRC was very weak (0.149, p-value = 0.001), while a weak positive correlation appeared with PRAD (0.270, p-value = 2.33E-08) and THCA (0.378. p-value = 6.65E-18). CD4+ T Cell had a positive correlation with COAD, HNSC, KIRC, READ, and THCA. The correlation was very weak with HNSC (0.179, p-value = 8.18E-18), KIRC (0.145, 0.002), and READ (0.182, 0.03). Additionally, CD4+ T Cells had a weak positive correlation with COAD (0.325, p-value = 2.38E-18) and a medium positive correlation with THCA (0.525, p-value = 5.74E-36). A very weak negative correlation appeared between CD4+ T cells with STAD (-0.165, p-value = 0.001). In CD8+ T cells, a positive correlation appeared with COAD, KIRC, PAAD, PRAD, READ, and UCEC, while a negative weak correlation was shown with THCA (0.341, p-value = 9.15E-15). The positive correlation was very weak with COAD(0.181, p-value = 2.42E-04), PAAD (0.181, p-value = 0.02), READ (0.204, p-value = 0.02), and UCEC (0.252, p-value = 1.49E-05), while a medium positive correlation appeared with PRAD (0.438, p-value = 6.78E-21). Dendritic cells showed a very weak positive correlation with BLCA (0.106, p-value = 0.04), GBM (0.150, p-value = 0.002), KIRC (0.154, p-value = 9.73E-04), and PAAD (0.156, p-value = 0.04), and a weak positive correlation with PRAD (0.301, p-value = 3.57E-10), READ (0.213, p-value = 0.01), THCA (0.244, p-value = 4.89E-08), and UCEC (0.230, p-value = 1.22E-04).

In contrast, a very weak negative correlation was shown between dendritic cells and STAD (-0.112, p-value = 0.03). Macrophages showed both positive and negative correlations with different types of cancer. The negative correlation was very weak with BLCA (-0.131, p-value = 0.012) and UCEC (-0.118, p-value = 0.044), and weak with STAD (-0.254, p-value = 6.95E-07). The positive correlation was very weak with HNSC (0.147, p-value = 0.001), KIRC (0.100, p-value = 0.04), and READ (0.192, p-value = 0.02), and a weak positive correlation was shown with COAD (0.252, p-value = 2.80E-07), PRAD (0.271, p-value = 2.00E-08), and THCA (0.311, p-value = 2.09E-12). Neutrophil had a very weak positive correlation with BLCA (0.107, p-value = 0.04), HNSC (0.107, p-value = 0.02), GBM (0.111, p-value = 0.02), and UCEC (0.177, p-value = 0.002), and a positive correlation appeared with COAD (0.244, p-value = 7.26E-07), KIRC (0.211, p-value = 5.11E-06), PRAD (0.282, p-value = 5.18E-09), and THCA (0.295, p-value = 3.02E-11). Purity showed both negative and positive correlations with many types of cancer. The negative correlation was very weak with COAD (-0.134, p-value = 0.007), PRAD (-0.124, p-value = 0.011), and READ (0.184, p-value = 0.03), and the positive correlation was weak with GBM (0.154, p-value = 0.001).

Survival analysis

Survival Analysis Using GEPIA

**Figure 13:**
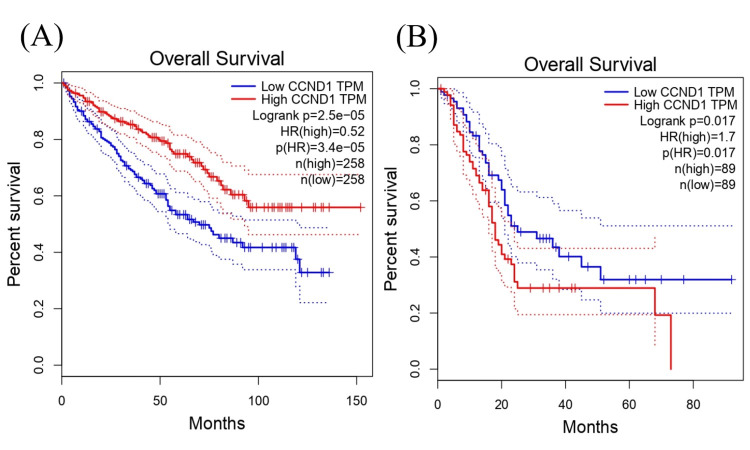
Using the GEPIA database, the relationship as shown by the hazard ratio and the log-rank p-value between the levels of CCND1 expression and patient survival outcomes in (A) KIRC and (B) PAAD.

The survival analysis revealed that the expression level of CCND1 in KIRC was associated with significant differences in survival with log-rank p-value = 2.5e−05. A hazard ratio of 0.52 indicated that the high-expression group had a lower hazard (or risk) of cancer (e.g., death) compared to the low-expression group, with a hazard ratio p-value of 3.4e-05 suggesting a protective effect. These findings provide insights into the potential prognostic value of CCND1 in KIRC (Figure 13A). Furthermore, a high expression of CCND1 in pancreatic PAAD was associated with a significantly higher hazard ratio compared with low expression groups; the log rank was 1.7, and the p-value was 0.017 (Figure 13B).

Cox Proportional Hazards Model Analysis

**Figure 14:**
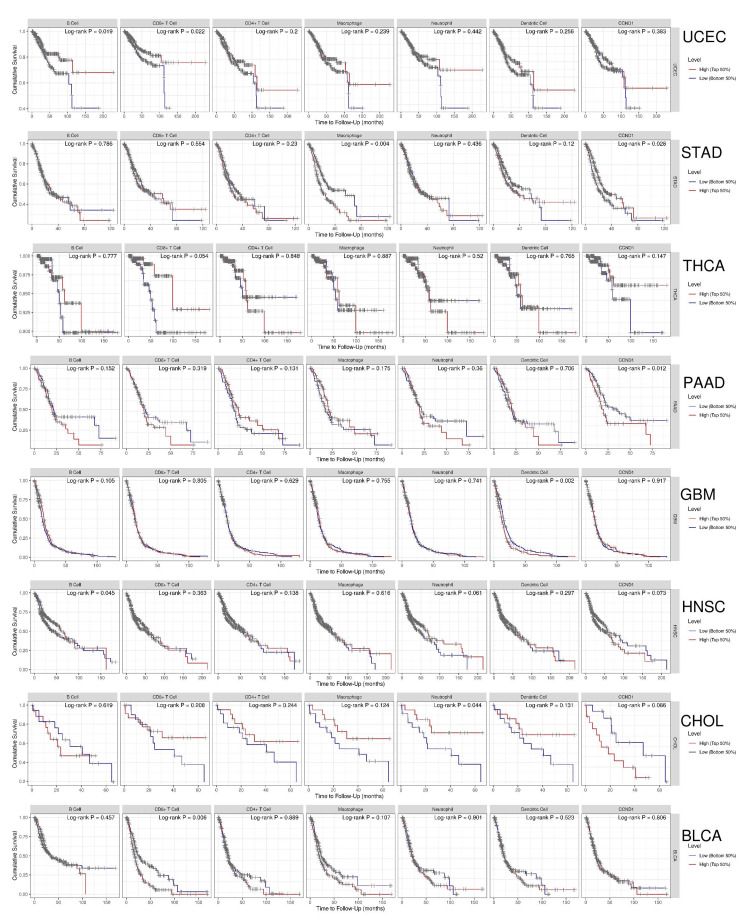
The data from TIMER includes Kaplan-Meier plots illustrating survival differences based on immune infiltrates and CCND1 gene expression among various cancers: UCEC, STAD, THCA, PAAD, GBM, HNSC, CHOL, and BLCA. We used the p-value of the log-rank test to compare the survival curves.

The interpretation of the survival analysis model from the TIMER dataset for different cancers using the provided coefficients and hazard ratios is stated here. Age was statistically significant in BLCA (375 patients with 165 dying), COAD (263 patients with 63 dying), GBM (504 patients with 429 dying), HNSC (408 patients with 180 dying), KIRC (486 patients with 156 dying), PAAD (170 patients with 90 dying), READ (82 patients with 14 dying), STAD (308 patients with 116 dying), and UCEC (488 patients with 83 dying), indicating that older age is associated with an increased hazard of these cancer types, in contrast to CHOL and PRAD, which revealed statistically insignificant effects. Gender, race, and purity did not individually demonstrate statistically significant effects on survival in all types of cancers. As indicated by Figure 14 and Table 3, there was a statistically significant correlation between the hazard ratio and immune cell infiltration as well as stage factors in many cancer types.

**Table 3:** The data from TIMER represents the Cox proportional hazards model of CCND1 for many cancer types, including BLCA, CHOL, COAD, ESCA, HNSC, KIRC, PAAD, READ, STAD, THCA, and UCEC. It illustrates how immune cell abundance and clinical factors affect the prognosis. Key factors such as age, cancer stages, and immune cell infiltrates (B cells, CD8+, CD4+, T cells,1` neutrophils, macrophages, and dendritic cells) are displayed in the table along with the associated p-values and hazard ratios. 95%CI_l: lower 95% confidence interval; 95%CI_u: upper 95% confidence interval

Cancer type	Parameter	Hazard ratio	(95% CI_l) - (95%CI_u)	p-value
BLCA	Age	1.031	1.014 - 1.048	0.000
	B Cell	0.033	0.002 - 0.69	0.028
	Macrophage	23.20	2.62 - 205.82	0.005
COAD	Age	1.040	1.017 - 1.065	0.001
	Stage4	7.746	2.27 - 26.44	0.001
ESCA	Stage3	3.972000e+00	1.16 - 1.360700e+01	0.028
	Stage4	1.654400e+01	3.87 - 7.072400e+01	0.000
GBM	Age	1.032	1.024 1.040	0.000
	Race (Caucasian)	2.194	1.02 - 4.71	0.044
	Dendritic	1.65	1.27 - 2.14	0.000
HNSC	Age	1.021	1.006 - 1.036	0.007
	Stage4	3.314	1.214 9.047	0.019
KIRC	Age	1.036	1.021 - 1.052	0.000
	Stage3	2.563	1.63 - 4.02	0.000
	Stage4	6.920	4.49 - 10.67	0.000
PAAD	Age	1.029	1.006 - 1.052000e+00	0.014
	CD4_Tcell	0.000	0.000 - 5.050000e-01	0.032
READ	Age	1.134000e+00	1.031 1.247000e+00	0.010
STAD	Age	1.04	1.02 - 1.06	0.000
	Stage3	3.18	1.49 - 6.80	0.003
	Stage4	5.66	2.08 - 15.41	0.001
	B cell	129.18	1.29 - 12872.57	0.038
	Macrophage	942.763	26.89 - 33044.54	0.000
UCEC	Age	1.039	1.016 - 1.062	0.001
	CD8_Tcell	0.005	0.000 - 0.401	0.018
	CD4_Tcell	0.000	0.000 - 0.357	0.024
CHOAL	Stage4	6.027000e+00	1.12 - 3.237600e+01	0.036
THCA	Age	1.155	1.084 - 1.230000e+00	0.000

Kaplan-Meier Plot Analysis

**Figure 15:**
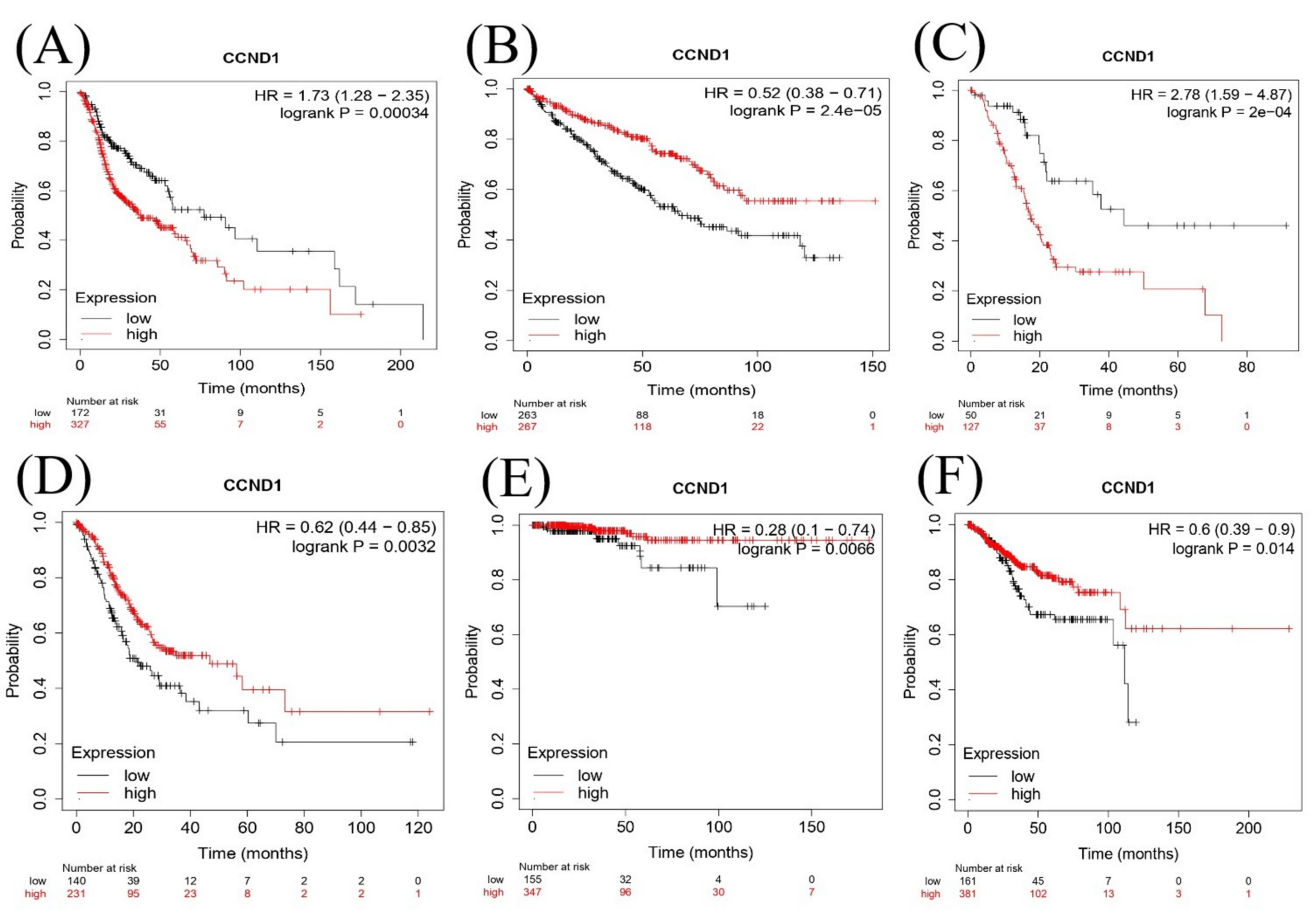
Kaplan-Meier survival plot describing CCND1 expression in (A) HNSC, (B) KIRC, (C) PAAD, (D) STAD, (E) THCA, and (F) UCEC.

We investigated the survival analysis using the Kaplan Meier Plotter, Cox proportional hazards model, and log-rank test to examine the relationship between CCND1 in different types of cancer as shown in (Figure 15A-15F). According to the Cox proportional hazards model and log-rank test p-value, there was a significant difference in survival between individuals with low and high expressions of CCND1 in HNSC with a p-value of 0.00034. The hazard ratio of 1.73 indicated a higher risk in the "high" group. KIRC and STAD had log-rank P = 2.4e−05 and 0.0032, respectively, and hazard ratios of 0.52 and 0.62, consequently, indicating that high expression of the CCND1 gene was associated with a lower risk of mortality. In contrast, PAAD showed that there was a significant difference in survival between low and high gene expressions with log-rank p = 2e-04 and a hazard ratio of 2.78, which suggested high risk in the high group. This indicated the prognostic or predictive value of CCND1 expression in KIRC. In the survival analysis of THCA and UCEC, the p-values were 0.0066 and 0.014., respectively, and the hazard ratios were 0.28 and 0.6, consequently. This showed that there was a significantly low risk of the event when there was low gene expression.

Genetic alteration analysis

Cancer Types Summary

**Figure 16:**
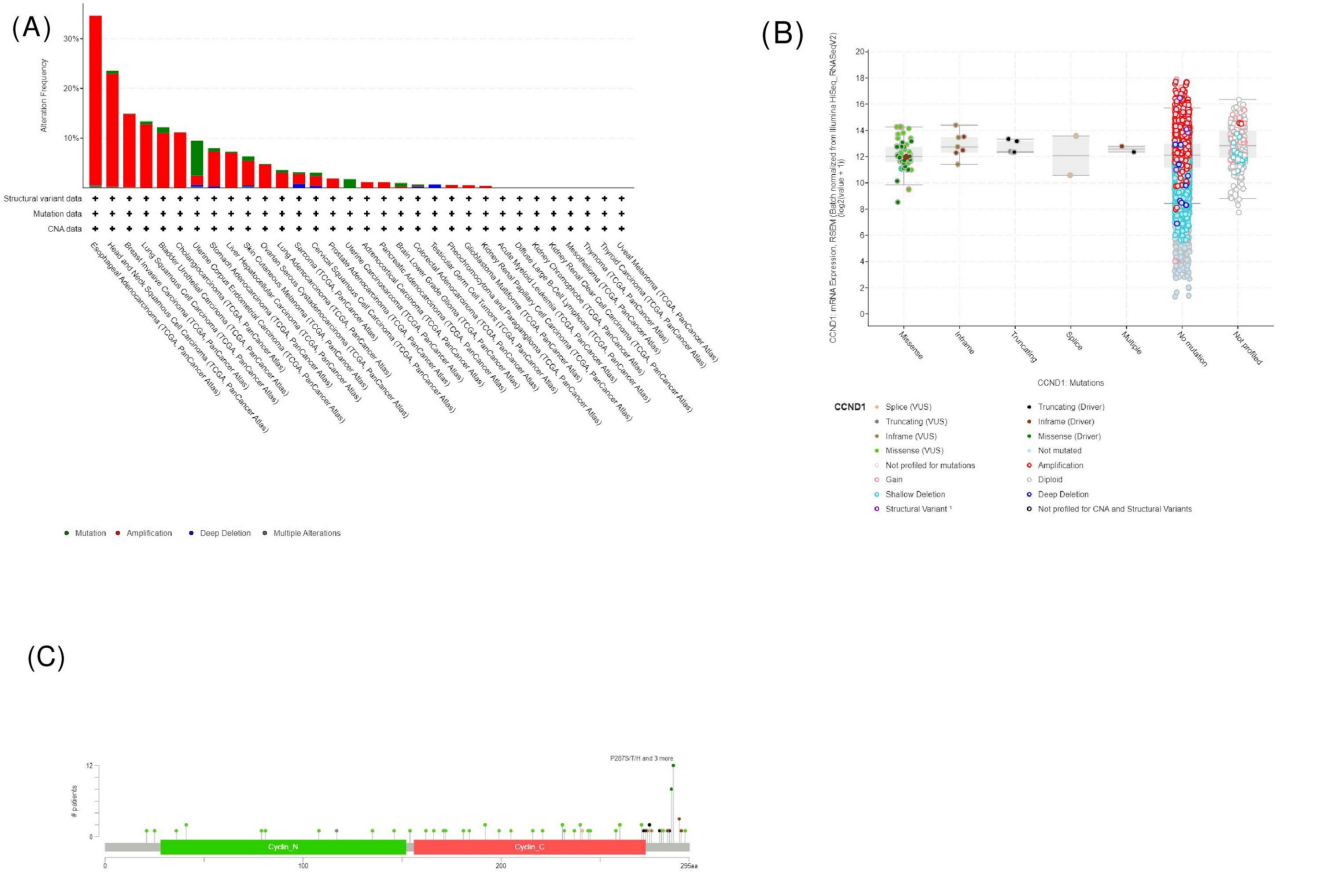
Genetic alteration analysis from the cBioPortal database. (A) The cancer types summary graph categorizes and summarises CCND1 alterations and the distribution of mutations across different cancer types. (B) Alteration of CCND1 mRNA expression according to the type of mutation. (C) The mutation frequency plot illustrates the occurrence of driver mutations and variants of uncertain significance (VUS) in CCND1 and the frequency and position of each mutation type.

We further explored the CCND1 genetic alteration status in human cancers in TCGA and Pan-Cancer Atlas cohorts using the cBioPortal database. As shown in Figure 16, in the total 10967 patient samples, 700 (6%) had a CCND1 genetic alteration. In most cancers, “amplification” was the primary genetic alteration type, and the highest amplification frequency of CCND1 (34.07%) occurred in cases with ESCA. Also, we have found in the chart in Figure 16A that the CCND1 gene was altered in these types of cancers, given here starting from cancers that represent the highest amplified samples: ESCA, HNSC, BLCA, CHOL, UCEC, STAD, PRAD, PAAD, and GBM. The percentage of altered samples according to the mentioned types is 34.62% of 182 ESCA cases, 23.52% of 523 HNSC cases, 12.17% of 411 BLCA cases, 11.11% of 36 CHOL cases, 9.45% of 529 UCEC cases, 7.95% of 440 STAD cases, 1.82% of 494 PRAD cases, 1.09% of 184 PAAD cases, and 0.51% of 592 GBM cases.

Furthermore, according to the chart shown in Figure 16A, types of gene alteration can be classified as follows.

Amplification: Based on the cancer types, there were 62 cases (34.07%), 119 cases (22.75%), 45 cases (10.95%), four cases (11.11%), 10 cases (1.89%), 31 cases (7.05%), nine cases (1.82%), two cases (1.09%), and three cases (0.51%) of amplified samples for ESCA, HNSC, BLCA, CHOL, UCEC, STAD, PRAD, PAAD, and GBM, respectively.

Mutation: There are three cases (0.57%), five cases (1.22%), 37 cases (6.99%), and three cases (0.68%) of mutated samples in HNSC, BLCA, UCEC, and STAD, respectively.

Multiple alterations: These occurred in ESCA, HNSC, and UCEC, with one case per type, 0.55%, 0.19%, and 0.19%, respectively.

Deep deletion mutations: These occurred in UCEC and STAD, with two cases (0.38%) and one case (0.23%), respectively.

mRNA Expression According to the Mutation Type

The plot in Figure 16B displays the change in CCND1 expression according to the type of mutation. If the patient had a missense mutation, the gene expression would be higher than if they had an inframe type, and if the patients had a splice mutation, they would have a gene expression lower than if they carried the previous types. The plot also shows that if the gene was not mutated, its expression would be at the highest level. In patients who had truncating or multiple mutations, their gene expression would be at the lowest level in comparison with all other types of mutation (Table 4). The not-profiled group shown in the graph shows patients whose data were not clear as to whether their genes were mutated or not. The plot in Figure 16C explains the frequency of each mutation type, which displays that missense mutation was the most dominant type. The plot shows that the most frequent mutation occurred at amino acid position 287. Of the 10,953 patients profiled, 12 had changes at this site, resulting in a shift from the original purine amino acid. The noticed alterations included serine, threonine, histidine, leucine, and arginine. The alteration could also happen in position 288 by the deletion of threonine amino acids.

**Table 4:** Distribution of CCND1 mutations categorized by mutation type and impact based on data from cBioPortal The somatic mutation frequency was 0.7%, driver mutation was 28, while the variants of uncertain significance (VUS) were 50.

Type of mutation	Driver 28	VUS 50
Missense	19	39
Truncating	5	1
Inframe	4	4
Splice	0	2
Fusion	0	4

Survival Analysis Using cBioPortal

The survival was compared across four categories: overall survival (OS), disease-specific survival (DSS), disease-free survival, and progression-free survival (PFS).

OS: The total number of patients included in the analysis was 46,206. The altered group consisted of 2,385 patients, while the unaltered group consisted of 43,821 patients. The median survival in the altered group was 39.66 months, with a 95% confidence interval (CI) of 36.76 to 45.39 months. The median survival in the unaltered group was 46.92 months, with a 95% CI of 46.06 to 47.90 months. The p-value was 5.98E-03, indicating a statistically significant difference in overall survival between the altered and unaltered groups. The q-value was 0.0239, suggesting that the difference in survival was unlikely to be a false positive.

DSS: The analysis included a total of 10,257 patients. Among them, 668 patients belong to the altered group, and 9,589 patients belong to the unaltered group. The median survival in the altered group was 146.50 months, with a 95% CI of 83.87 to NA (not available). The median survival in the unaltered group was 133.74 months, with a 95% CI of 117.40 to 162.08 months. The p-value was 0.319, indicating no statistically significant difference in disease-specific survival between the altered and unaltered groups. The q-value was 0.638, further supporting the absence of a significant difference.

Disease-free survival: The analysis included a total of 5,383 patients. Among them, 390 patients belonged to the altered group, and 4,993 patients belonged to the unaltered group. Median survival values were not available for both the altered and unaltered groups. The p-value of 0.626 indicated no statistically significant difference in disease-free survival between the altered and unaltered groups. The q-value was 0.834, further confirming the absence of a significant difference.

PFS: The analysis included a total of 10,846 patients. Among them, 695 patients belonged to the altered group, and 10,151 patients belonged to the unaltered group. The median survival in the altered group was 66.84 months, with a 95% CI of 46.85 to NA. The median survival in the unaltered group was 58.95 months, with a 95% CI of 55.53 to 65.00 months. The p-value was 0.904, indicating no statistically significant difference in progression-free survival between the altered and unaltered groups. The q-value of 0.904 further reinforced the absence of a significant difference.

**Figure 17:**
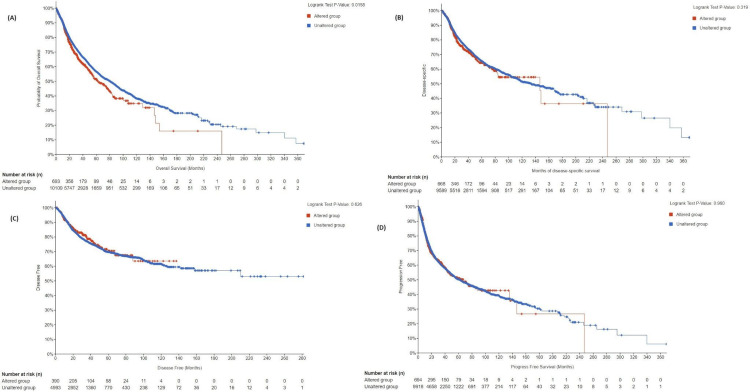
Kaplan-Meier survival curves from the cBioPortal database compare survival rates between groups with and without CCND1 mutations across four different categories. (A) Overall survival, (B) disease-specific survival, (C) disease-free survival, and (D) progression-free survival.

The main results suggested that the overall survival was statistically significant between altered groups and unaltered groups, and the unaltered groups had higher median survival. Nonetheless, no significant differences were noted between the two groups in terms of disease-specific, disease-free, and progression-free survival, as shown in Figure 17.

Validation

**Figure 18:**
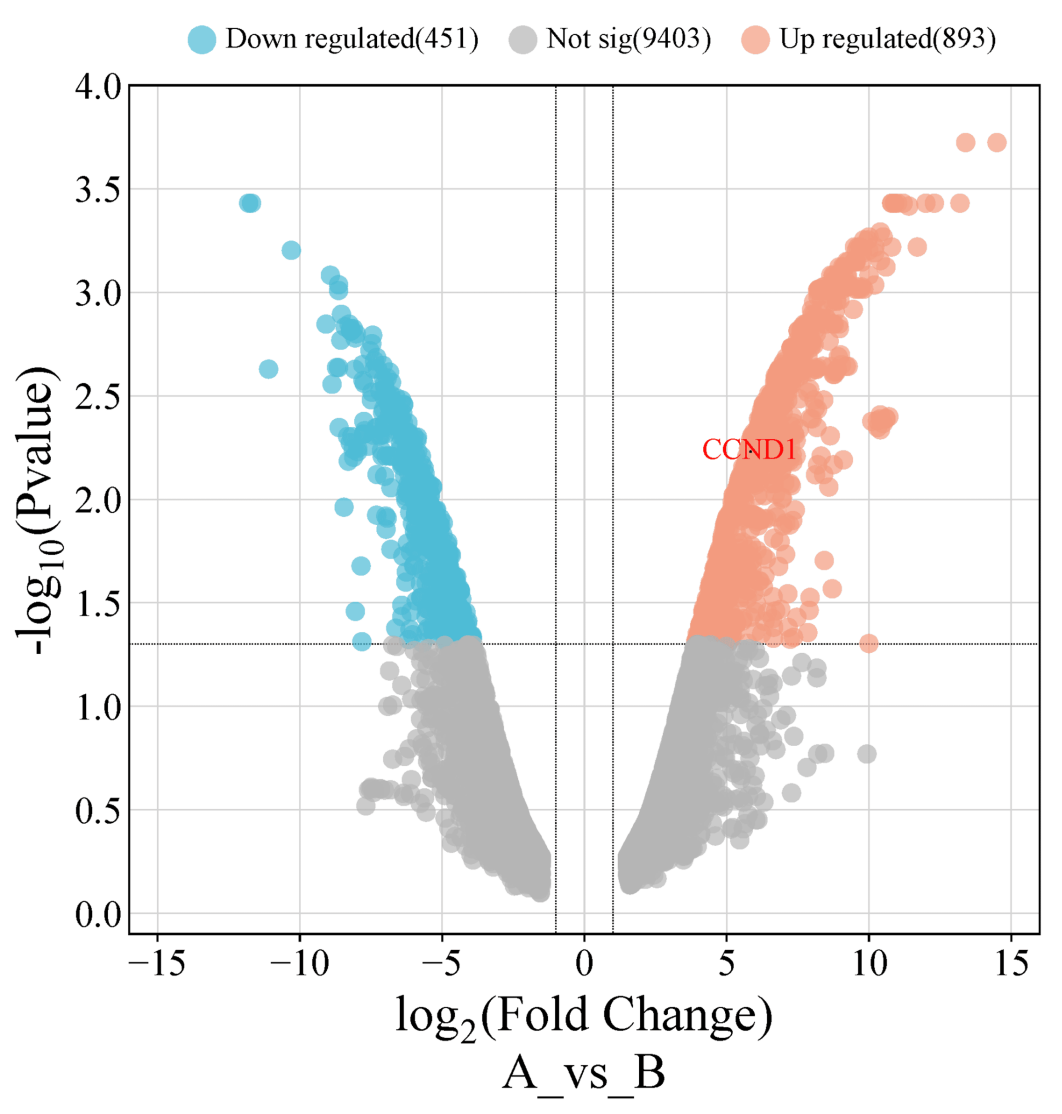
A volcano plot visualizes the validation data of the GSE40185 dataset from the GEO NCBI database, and shows the CCND1 expression level in HNSC.

After conducting a comprehensive analysis of CCND1 mRNA expression using three different databases (GEPIA, UALCAN, and TIMER), as well as a survival analysis using the GEPIA and Kaplan-Meier plotter databases, the results showed that CCND1 expression was significantly upregulated in six cancer types (HNSC, KIRC, PAAD, STAD, THCA, and UCEC). Thereafter, we investigated these findings in two online validating databases. The first database (GSE40185), GEO, revealed that CCND1 was significantly upregulated in HNSC, with an adjusted p-value of 0.0059 and logFC = 5.84. The volcano plot for these cancers was created using http://bioinformatics.com.cn/, as seen in Figure 18.

UCSC Xena database

**Figure 19:**
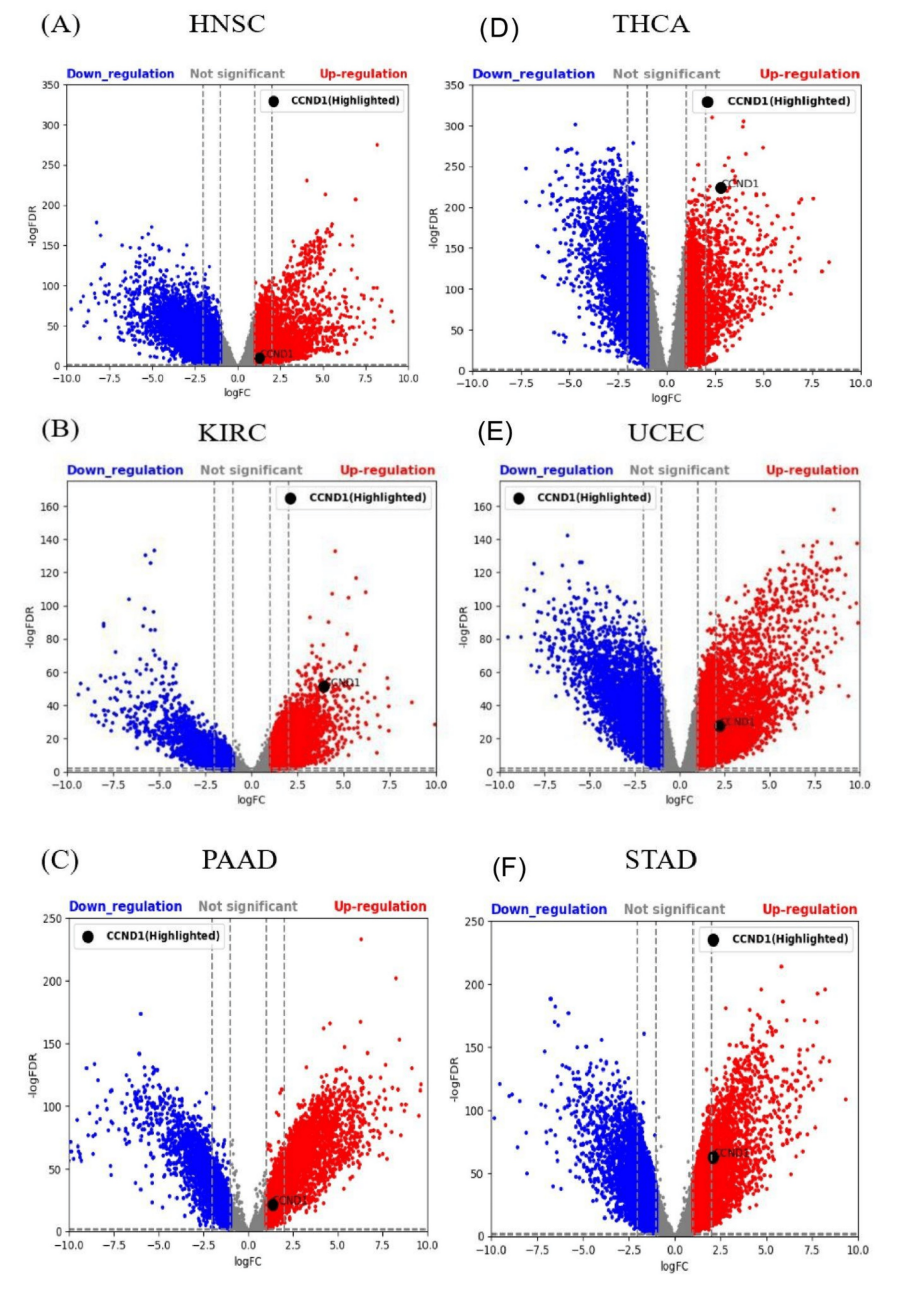
A volcano plot visualizes the validation data from the UCSC Xena dataset. (A-F) CCND1 expression in HNSC, KIRC, PAAD, THCA, UCEC, and STAD; GTEx represents a normal tissue sample vs TCGA, which represents a cancer tissue sample.

The GTEx and TCGA samples in HNSC, KIRC, PAAD, STAD, THCA, and UCEC were 55 and 520, respectively, 28 and 531, respectively, 167 and 179, respectively, 174 and 414, respectively, 279 and 512, respectively, and 78 and 181, respectively, respectively. CCND1 expression using LogFC, p-values, and adjusted p-values was statistically significant in HNSC, KIRC, PAAD, STAD, THCA, and UCEC, as shown in Figure 19A-19F.

## Discussion

Cyclin D1 is a 36-kDa protein encoded by the CCND1 gene, located on chromosome 11q13. Cyclin D1 is expressed by the majority of normal human cells, apart from cells derived from bone marrow stem cell lines [19]. A pan-cancer analysis of CCND1 may discover tumor diagnostic and prognostic biomarkers and the distinctive and similar characteristics that distinguish genes among multiple tumors [20,21,22].

In the current study, we performed a pan-cancer investigation of CCND1 expression, methylation, prognosis, immune infiltrations, genetic alteration, survival, and differentially expressed genes (DEGs) across tumors using GEPIA, UALCAN, TIMER, GSCA, cBioPortal, GEO, and UCSC Xena databases, and Kaplan-Meier plot. While CCND1 was expressed at relatively small levels in normal tissues, it was substantially expressed in BLCA, CHOL, COAD, ESCA, GBM, HNSC, KIRC, PAAD, RRAD, READ, STAD, THCA, and UCEC (Figures 1, 2, 4). In stages, CCND1 revealed statistical significance between normal and all stages in COAD, ESCA, KIRC, READ, STAD, and THCA (Figure 8 and Table 1), and it could be used as a diagnostic biomarker in these types of cancer. The survival analysis revealed that a high expression of CCND1 was a poor prognosis in HNSC and PAAD, while a high expression of CCND1 was a good prognosis in KIRC, STAD, THCA, and UCEC (Figures 9, 10). According to previous studies that support our findings, CCND1 overexpression in pancreatic adenocarcinoma correlates with aggressive features and poorer survival, indicating its potential as an adverse prognostic factor [23].

Furthermore, CCND1 overexpression in HNSC is significantly associated with higher tumor stage and lymph node metastasis, suggesting its potential as a prognostic marker [24]. In addition, clear cell renal cell carcinoma (ccRCC) reveals low CCND1 correlates with adverse outcomes and suggests a unique prognostic role in ccRCC [19]. Contrary to our findings, a study revealed that reduced overall and progression-free survival is linked to overexpression of CCND1 in gastric cancer and also linked to poor differentiation and negative erb2 status, emphasizing its prognostic significance in gastric cancer [25]. Additionally, a study discovered that the overexpression of CCND1 in papillary thyroid carcinoma (PTC) is linked to the aggressiveness and recurrence of the illness. Reduced disease-free survival is correlated with elevated CCND1 levels and increased C-myc expression, indicating a possible function for these markers as prognostic indicators in PTC [26]. Moreover, CCND1 overexpression in endometrial lesions is substantially linked to endometrioid carcinoma, clear cell carcinoma, and atypical complex hyperplasia. A higher risk of metastasis and a poor outcome are correlated with high CCND1 expression. It has limited effectiveness in differentiating between neoplastic and non-neoplastic lesions, despite its promise as a prognostic marker [27].

Gene expression is controlled by a chemical alteration called DNA methylation. In tumor cells, there is aberrant DNA methylation that mainly targets CpG islands in regulatory elements of gene expression [28,29]. Our result revealed that there was a negative correlation between the CCND1 gene's methylation levels and its expression in multiple types of cancer, particularly in ESCA, HNSC, and STAD. This suggests that CCND1 methylation may have a role in regulating the expression of the CCND1 gene in these cancers. The survival analysis in BLCA showed reduced DSS with decreased methylation of CCND1. A lower methylation of CCND1 in PRAD was associated with worse PFS and DFI. Conversely, higher methylation levels of CCND1 in UCEC and STAD correlated with an increased risk of mortality, especially in DSS and DFI, respectively. The differential methylation analysis of CCND1 demonstrated a considerable downregulation in several cancers, including COAD, KIRC, and THCA, with COAD showing the most significant downregulation. These results point to a possible involvement of CCND1 methylation in the pathogenesis of these cancers (Figure 11).

The OS of CCND1 revealed overexpression associated with reduced median survival in an altered group (Figure 17A). The results of our study showed that the "amplification" of CCND1 alterations was mostly seen in ESCA, HNSC, BLCA, and CHOL (Figure 16). CCND1 expression significantly impacts patient prognosis in various cancers, such as BLCA, COAD, GBM, HNSC, KIRC, PAAD, READ, STAD, and UCEC, by influencing immune cell infiltrations. Older age increases the death risk for many types of cancer. Stage 4 is associated with elevated risk in CHOL, COAD, KIRC, and STAD. CD4+ and CD8+ T cells decrease death risk. Macrophages increase risk in BLCA and STAD, while CCND1 presence decreases risk in KIRC. Various factors contribute differently to death risk across cancer types (Table 3). 

## Conclusions

In conclusion, CCND1 is a promising biomarker for prognosis and diagnosis in many cancer types. CCND1 expression was found to differ significantly between normal and malignant tissues in all stages of COAD, ESCA, KIRC, READ, STAD, and THCA, according to our pan-cancer research, suggesting that CCND1 may be used as a diagnostic biomarker in these types of cancer. These results imply that CCND1 levels may help in the early identification and detection of these malignancies. Furthermore, in HNSC, KIRC, and PAAD, CCND1 exhibits promise as a predictive biomarker. Additionally, a negative association was found between the expression of CCND1 and methylation in several types of tumors, particularly ESCA, HNSC, and STAD, according to our analysis of CCND1 methylation patterns. This shows that CCND1 expression in these malignancies may be regulated by CCND1 methylation.

Furthermore, downregulation of CCND1 was found by differential methylation analysis in COAD, KIRC, and THCA, indicating a possible involvement of CCND1 methylation in the etiology of these cancers. Moreover, a negative association was found between CCND1 methylation levels and expression in STAD, ESCA, and head and neck HNSC. These CCND1 methylation patterns may also be used as a diagnostic and prognostic sign in these types of cancer. While higher methylation levels of CCND1 correlate with an increased risk of mortality, particularly in DSS and DFI in UCEC and STAD, respectively, lower methylation of CCND1 is linked to worse PFS and disease-free interval DFI in PRAD. It is crucial to acknowledge the limitations of this study, namely the absence of validation through wet lab trials. Further research employing experimental validation methods, like web-lab approaches, is necessary to validate the diagnostic and prognostic significance of CCND1 in various types of tumors.

## Appendices

**Table 5:** Abbreviations

Abbreviation	Full form
BLCA	Bladder urothelial carcinoma
BRCA	Breast invasive carcinoma
CHOL	Cholangiocarcinoma
COAD	Colon adenocarcinoma
DLBC	Lymphoid neoplasm diffuse large B-cell lymphoma
GBM	Glioblastoma multiforme
ESCA	Esophageal carcinoma
HNSC	Head and neck squamous cell carcinoma
KIRC	Kidney renal clear cell carcinoma
LAML	Acute myeloid leukemia
LGG	Brain lower-grade glioma
OV	Ovarian serous cystadenocarcinoma
PAAD	Pancreatic adenocarcinoma
PRAD	Prostate adenocarcinoma
PCPG	Pheochromocytoma and paraganglioma
READ	Rectum adenocarcinoma
STAD	Stomach adenocarcinoma
THCA	Thyroid carcinoma
THYM	Thymoma
UCEC	Uterine corpus endometrial carcinoma
UCS	Uterine carcinosarcoma
OS	Overall survival
DSS	Disease-specific survival
DFI	Disease-free interval
PFS	Progression-free survival
TIMER	Tumor Immune Estimation Resource
GEPIA	Gene Expression Profiling Interactive Analysis
UALCAN	University of ALabama at Birmingham CANcer data analysis portal
GSCA	Gene Set Cancer Analysis
GEO	Gene Expression Omnibus
TCGA	The Cancer Genome Atlas
GTEx	Genotype-Tissue Expression
UCSC	University of California Santa Cruz
